# The Complete Mitochondrial Genome of *Conopomorpha sinensis* (Lepidoptera: Gracillariidae) Sample from Taiwan

**DOI:** 10.3390/genes17050594

**Published:** 2026-05-21

**Authors:** Yu-Yun Kuo, Tai-Chuan Wang, Pin-Chang Chen, JenYu Chang, Yu-Shin Nai

**Affiliations:** 1 Department of Entomology, National Chung Hsing University, Taichung City 40227, Taiwan; yunhallo113gif@gmail.com (Y.-Y.K.); 3121531ttl78@gmail.com (P.-C.C.); 2 Laboratory of Plant Protection, Crop Environment Section, Kaohsiung District Agricultural Research and Extension Station, Ministry of Agriculture, Pingtung City 908126, Taiwan; insect6925@mail.kdais.gov.tw; 3 Agricultural Chemistry Division, Taiwan Agricultural Research Institute, Ministry of Agriculture, Taichung City 413008, Taiwan; jychang@tari.gov.tw

**Keywords:** mitogenome, Gracillariidae, *Conopomorpha sinensis*, litchi pest, longan pest

## Abstract

Background: The litchi fruit borer, *Conopomorpha sinensis* (Lepidoptera: Gracillariidae), is a devastating pest affecting litchi and longan production across Asia. Although a reference mitochondrial genome (mitogenome) has been published, its utility is limited by the lack of precise geographical data and raw sequencing data. Methods: In this study, we sequenced and characterized the complete mitogenome of *C. sinensis* collected from Taiwan using a hybrid assembly of Illumina and Oxford Nanopore technologies. Results: The assembled mitogenome is 17,301 bp in length with a mean sequencing depth of 19,155-fold, comprising 13 protein-coding genes (PCGs), 22 transfer RNA genes, two ribosomal RNA genes, and an AT-rich control region. Notably, we identified a rare tRNA gene rearrangement (*trnR-trnA-trnN-trnS1-trnE-trnF*) that deviates from the ancestral lepidopteran ditrysian pattern. Comparative analysis revealed a 94.65% overall sequence identity with the reference mitogenome, though the PCGs remained highly conserved at 99.35%. Variant analysis demonstrated that this divergence is predominantly driven by structural variations (228 indels) rather than nucleotide substitutions (2 SNPs) across the entire mitogenome; furthermore, 94.7% of the indels were identified in the control region and intergenic spacers. Subtle differences in codon usage were also observed in the *ND6* start codon (ATT vs. ATA) and *COX1* stop codon (TAA vs. T). Phylogenetic and molecular clock analyses robustly clustered the Taiwan specimen within the *C. sinensis* clade. Molecular dating estimates that the *Conopomorpha* lineage originated during the Late Cretaceous (~77.23 Ma). Notably, the divergence between the Taiwan specimen and the reference lineage was estimated to be negligible (<0.01 Ma) within the protein-coding regions, demonstrating a high degree of purifying selection that maintains coding-sequence stability across geographically distinct specimens, even as substantial variation accumulates in non-coding genomic regions. Conclusions: These findings provide high-resolution genomic resources and a temporal framework for the evolutionary study of Gracillariidae, offering foundational tools for targeted pest management.

## 1. Introduction

**Figure 1:**
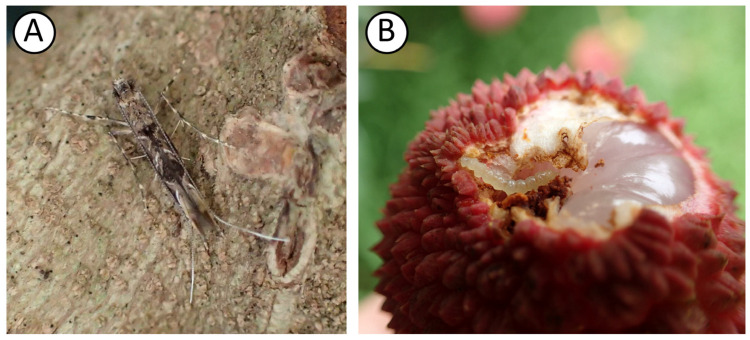
Photographs of live *C. sinensis* (photo credit: Tai-Chuan Wang). (**A**) An adult at rest, showing sword-shaped forewings marked with yellowish-white wavy lines forming zigzag pattern. (**B**) A larva infesting a litchi fruit, with a yellowish-brown head and white abdomen.

*Conopomorpha sinensis* Bradley, 1986 (Lepidoptera: Gracillariidae), known as the litchi fruit borer (LFB), is a major pest of litchi (*Litchi chinensis*) and longan (*Dimocarpus longan*) in Taiwan [1]. It is also found in Nepal, Vietnam, China, Thailand, India, and Southeast Asia [2,3]. The adult *C. sinensis* measures approximately 4.3 × 0.6 mm, with a wingspan of about 10 mm [4]. Its head is grayish-white with prominent black compound eyes. The antennae are filiform and longer than the body. The thorax is grayish-yellow, while the forewings are brown, sword-shaped, and marked with yellowish-white wavy lines forming a zigzag pattern at rest. The hindwings are narrow, elongated, and tapering, with long fringe scales along the margins (Figure 1A) [3,4,5]. During the fruiting season of litchi and longan, *C. sinensis* completes approximately four to five generations in the field [4]. The larvae feed on shoots, leaves, flower buds, and fruit, causing significant fruit drop during the mid-to-late stages of fruit development (Figure 1B) [5]. Larval infestation in fresh fruits reduces fruit yield and affects quality, leading to substantial economic losses in the litchi and longan industries [3,5,6,7].

Although a mitogenome sequence for *C. sinensis* has been previously deposited in GenBank (Accession: OK310517), while its reliability for comparative studies is hindered due to the absence of associated raw sequencing data in the NCBI Sequence Read Archive (SRA) and lack of precise metadata, such as GPS coordinates. Our systematic search of the SRA database using the taxon ID for *C. sinensis* (txid: 940481) confirmed that no datasets corresponding to the previous record are currently available. To address these limitations, a complete and high-quality mitogenome of *C. sinensis* sampled from Taiwan was presented. By making the raw sequencing data publicly available and providing precise geographical documentation, this study not only rectifies the limitations of previous records but also provides a traceable and reliable reference for the molecular identification study of Gracillariidae. These data will serve as a foundational tool for future research into the pest’s population structure and the development of more targeted molecular-based management strategies.

## 2. Materials and Methods

### 2.1. Sample Collections and Identification

Larvae in dropped litchi fruits were collected from Dashu District, Kaohsiung City, Taiwan (22°43′04.3″ N 120°25′56.6″ E). The sample was deposited at the Insect Pathology and Genomics Laboratory (IPL) in the Department of Entomology, National Chung Hsing University (Yu-Shin Nai, ysnai@nchu.edu.tw), under the voucher number NCHU IPL 74.

To identify the collected larvae, we consulted the established records of lepidopteran pests associated with longan and lychee in Taiwan. Historical surveys indicated that gracillariid larvae infesting dropped litchi fruits in Taiwan are exclusively *C. sinensis*, whereas the sympatric species *C. litchiella* feeds mainly on the shoots rather than the fruits [8]. Furthermore, *C. cramerella* (the cocoa pod borer) has not been reported in Taiwan [9]. To supplement these ecological and geographical identifications, molecular verification was performed using the cytochrome c oxidase subunit 1 (*COX1*) barcode region. Phylogenetic analysis (Supplementary Table S1) confirmed that the *COX1* sequence of our sample clusters with high statistical support within the *C. sinensis* clade. Consequently, based on these ecological and geographical records, all larvae examined in this study were identified as *C. sinensis*.

### 2.2. DNA Extraction, Genomic Sequencing, and Data Analysis

Genomic DNA (gDNA) was extracted from five individuals using QIAGEN Genomic-tip 20/G Kit (QIAGEN, Hilden, Germany). Sequencing was performed on both Illumina (San Diego, CA, USA) NovaSeq X Plus with 151 bp paired-end sequencing and Oxford Nanopore Technologies (ONT) sequencing. Illumina reads were trimmed using TrimGalore v0.6.7 to remove adapters and reads with a Phred score below 20 [10], while ONT reads were processed using Dorado v7.3.11 (https://github.com/nanoporetech/dorado, accessed on 18 May 2026). Specifically, basecalling was performed with the high-accuracy (hac) model. The SQK-NBD114-96 kit (Oxford Nanopore Technologies, Oxford, UK) configuration was utilized for integrated demultiplexing and high-precision adapter trimming. Subsequent read quality assessment was conducted via FastQC v0.11.9 (https://www.bioinformatics.babraham.ac.uk/projects/fastqc/, accessed on 18 May 2026) for short reads and NanoPlot v1.42.0 [11] for long reads using default parameters (Supplementary Figure S1). Clean reads were mapped to the reference mitogenome (*C. sinensis* GenBank: OK310517) using BWA v0.7.17 (short reads) [12] and minimap2 v2.24 (long reads) [13]. Hybrid assembly was conducted with Unicycler v0.4.8. [14]. Sequencing depth was calculated using SAMtools v1.21 depth [15], and annotation was performed using MITOS2 v2.1.9. [16,17] to identify coding DNA sequence (CDS), transfer RNA (tRNA), ribosomal RNA (rRNA), and the control region.

### 2.3. Phylogenetic Analysis

Phylogenetic analysis was conducted using two datasets from NCBI database: (1) a mitogenome dataset comprising representatives from nine Gracillariidae species as the ingroup, five Psychidae species, four Tineidae species, and one Meessiidae species serving as outgroups (Supplementary Table S2) [3,18,19,20,21,22,23,24,25,26,27,28,29,30,31,32]; *Bombyx mori* (Bombycidae, Bombycoidea: GenBank: AF149768) was used to root this phylogenetic tree. (2) *COX1* gene database containing 25 sequences from Australia, India, Vietnam, Malaysia, China, Taiwan, USA, Malay Archipelago and Papua New Guinea [3,33,34,35,36,37,38,39,40] to reconfirm species identity (Supplementary Table S1). Sequences were aligned with MUSCLE v5.1 [41]. Phylogenetic trees were reconstructed using IQ-TREE2 [42,43] and MrBayes v3.2.7a [44,45,46] with the best-fit model (GTR + R4 for ML; GTR + γ for BI), which were selected using ModelFinder v2.4.0 [47,48] and ModelTest-NG v0.1.7 [47] for mitogenome and *COX1* databases, respectively, following the procedure from a previous study [42].

### 2.4. Divergence Time Estimation

The divergence times of *C. sinensis* and its related lineages were estimated using the RelTime method [49,50] implemented in MEGA11 [51]. The analysis was conducted based on a concatenated supermatrix of 13 PCGs, across 20 taxa, resulting in a final alignment length of 11,097 bp. Following codon-based alignment via the MUSCLE algorithm, gene alignments were manually inspected to ensure accuracy. Ambiguously aligned regions and excessive gaps at the 5′ and 3′ termini were trimmed to preserve the integrity of the phylogenetic signal.

The trimmed sequences were subsequently concatenated, and the GTR + I + G4 substitution model was identified as the best-fit model by ModelFinder v2.4.0 [47,48]. An initial phylogenetic framework was reconstructed using RAxML-NG v. 1.2.2 [52] via the Maximum Likelihood (ML) approach. This ML topology served as the starting tree for molecular clock analysis. Molecular dating was calibrated using three secondary calibration points retrieved from TimeTree5 [53]. To account for temporal uncertainty, each calibration point was constrained with a uniform distribution (±10% of adjusted time):

1. The Most Recent Common Ancestor (MRCA) of the genus *Phyllonorycter* (median: 22.0 Ma; range: 19.8–24.2 Ma);
2. The MRCA of *Phyllonorycter* and *Cameraria* (median: 57.0 Ma; range: 51.3–62.7 Ma);
3. The MRCA of the clade comprising *Phyllonorycter*, *Cameraria*, and *Phyllocnistis citrella* (median: 76.0 Ma; range: 68.4–83.6 Ma).

The final chronogram was inferred with an estimated log-likelihood of—120,410.40, and the divergence times were annotated at the respective nodes.

### 2.5. Mitogenomic Comparison and Variant Identification

Whole-mitogenomic alignment between the Taiwanese specimen (PV603655) and the reference sequence (OK310517) was performed using the dnadiff v1.3 utility within the MUMmer package [54]. To determine the regional distribution of genomic variation, these variants were mapped against the annotation of the mitogenome of Taiwanese specimen using bedtools v2.31.1 [55]. This process facilitated the quantification of variants within specific genomic features, including protein-coding genes (PCGs), transfer RNA (tRNA), ribosomal RNA (rRNA), the control region, and intergenic spacer.

## 3. Results

### 3.1. Mitogenome Assembly

**Figure 2:**
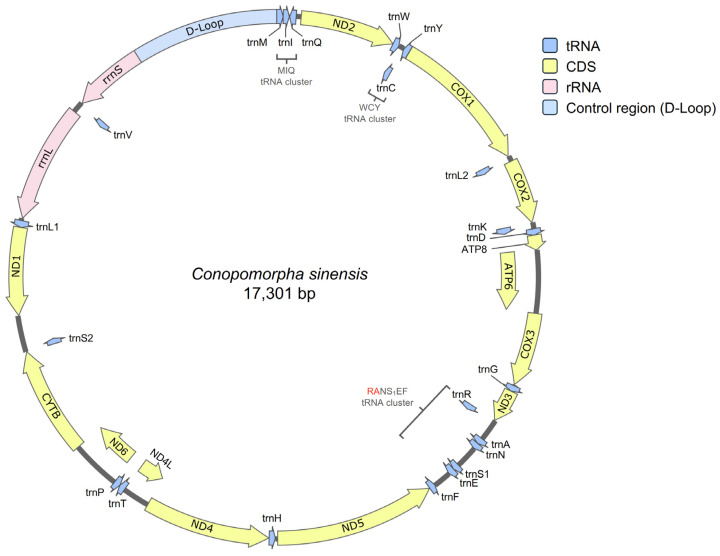
The complete mitogenome of *C. sinensis* (Bradley, 1986). The genome is 17,301 bp in length and contains 22 tRNAs, 2 rRNAs, 13 CDS, and an AT-rich control region.

By leveraging a hybrid assembly approach that combines Illumina short-reads and ONT long-reads, we successfully resolved the complete mitogenome of *C. sinensis* from Taiwan (17,301 bp; GenBank: PV603655). This strategy ensured high base-call accuracy and the structural integrity of the repetitive AT-rich control regions, which are often truncated in short-read-only assemblies. The exceptionally high sequencing depth (19,155×) further validates the robustness of the consensus sequence (Figure 2 and Supplementary Figure S2). The mitogenome consisted of 13 protein-coding genes, 22 transfer RNA genes, 2 ribosomal RNA genes, and a major non-coding AT-rich control region (Figure 2 and Table 1). The overall nucleotide composition was 42.23% A, 9.55% C, 6.81% G, and 41.41% T, with an AT content of 83.64% and GC content of 16.36%. Compared with the previously published mitogenome of *C. sinensis* (GenBank: OK310517), the Taiwan sample showed 94.65% overall sequence identity, and the protein-coding regions were more conserved, with a sequence identity of 99.35%, indicating the same species but reflecting geographic differentiation between isolates [56].

### 3.2. Protein-Coding Genes

There was a total of thirteen PCGs found in the *C. sinensis* mitogenome. The 13 PCGs exhibited a high level of conservation in their amino acid sequences; however, divergent synonymous codon usage was observed. Notably, the Taiwan isolate utilizes a complete TAA stop codon for *COX1*, in contrast to the truncated T stop codon in the reference genome (OK310517). Furthermore, the *ND6* gene in the Taiwan specimen initiates with ATT, differing from the ATA start codon recorded in the reference. Such variations in initiation and termination signals are indicative of subtle micro-evolutionary shifts between geographically isolation.

### 3.3. Transfer RNA and Ribosomal RNA Genes

The mitogenome of *C. sinensis* contained twenty-two tRNA and two rRNA genes (Figure 2 and Table 1). A significant finding in the tRNA organization was the rearranged RANS1EF cluster located between 5928 and 6925 bp. This arrangement (*trnR-trnA-trnN-trnS1-trnE-trnF*) deviates from the ancestral lepidopteran ditrysian pattern. The high sequencing depth (average sequencing depth between *trnR* to *trnF* was 9935.9×) and long-read evidence from this study provide unambiguous confirmation of this rearrangement, offering a more reliable structural reference than previous records lacking raw data support.

### 3.4. Phylogenetic Analysis

**Figure 3:**
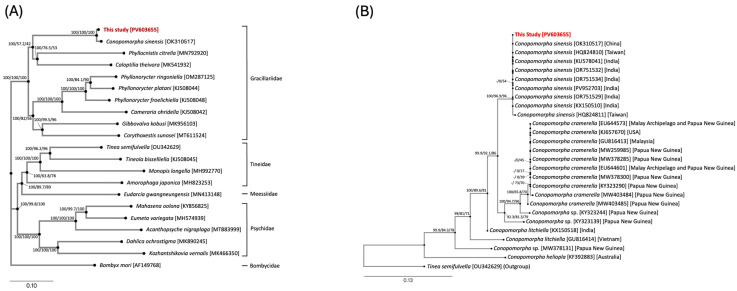
Phylogenetic analysis of *C. sinensis.* (**A**) Phylogeny based on whole mitogenomes (excluded AT-rich control region) of 19 species from the superfamily Tineidae. The tree includes 9 species from Gracillariidae: *C. sinensis* [OK310517] [3], *Caloptilia theivora* [MK541932] [23], *Cameraria ohridella* [KJ508042] [19], *Gibbovalva kobusi* [MK956103] [22], *Corythoxestis sunosei* [MT611524] [27], *Phyllocnistis citrella* [MN792920] [26], *Phyllonorycter ringoniella* [OM287125] [30], *Phyllonorycter platani* [KJ508044] [19], and *Phyllonorycter froelichiella* [KJ508048] [19]; 5 species from Psychidae: *Mahasena oolona* [KY856825] [20], *Eumeta variegata* [MH574939] [21], *Acanthopsyche nigraplaga* [MT883999] [29], *Dahlica ochrostigma* [MK890245] [24], and *Kozhantshikovia vernalis* [MK466350] (Unpublished); 4 species from Tineidae: *Tinea semifulvella* [OU342629] [31], *Tineola bisselliella* [KJ508045] [19], *Monopis longella* [MH992770] [28], and *Amorophaga japonica* [MH823253] [25]; one species from Meessiidae: *Eudarcia gwangneungensis* [MN413148] [32]. *Bombyx mori* [AF149768] [18] serves as the outgroup. (**B**) Phylogenetic trees of *Conopomorpha* species based on *COX1* sequences. The analysis including 25 *Conopomorpha* sequences and *Tinea semifulvella* as the outgroup. The sample from this study [PV603655] clustered within the *C. sinensis* clade with high statistical support. Trees were reconstructed using MrBayes (BI) and IQ-TREE (ML). Nodal support values are shown as Posterior Probability (PP) (for BI), UFBoot2 (for ML), and SH-aLRT (for ML) (PP/UFBoot2/SH-aLRT).

Despite the 5.35% mitogenomic divergence between specimens from Hainan Island, China (OK310517), and Taiwan (this study), phylogenetic analysis based on the complete mitogenome of ten Gracillariidae species indicated that the Taiwan sample of *C. sinensis* clusters most closely with *C. sinensis* (GenBank: OK310517), with 100% bootstrap support (Figure 3A). This result is also supported by the phylogenetic analysis based on *COX1* sequences (Figure 3B).

### 3.5. Divergence Time Estimation

**Figure 4:**
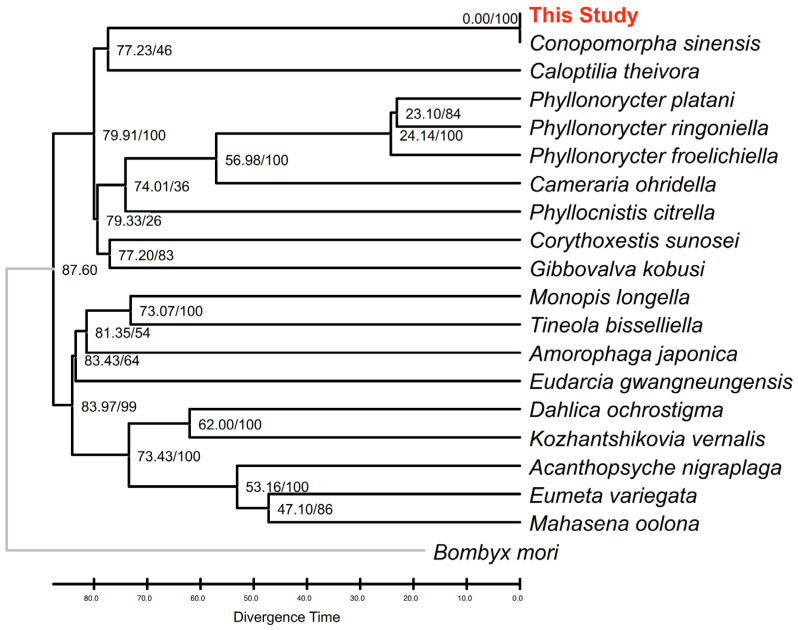
Chronogram of *C. sinensis* and related lineages based on divergence time estimation. The evolutionary timeframe was inferred using the RelTime ML method in MEGA11, utilizing a concatenated dataset of 13 mitochondrial PCGs. The topology was reconstructed via RAxML-NG under the GTR + I + G4 substitution model. Numbers at the nodes indicate the estimated divergence times in million years ago (Ma), followed by bootstrap support values (Time/BS). The horizontal scale bar at the bottom represents time in Ma. *Bombyx mori* was employed as the root for the phylogenetic framework. Nodes labeled with 0.00 represent an estimated divergence time of <0.01 Ma, reflecting negligible genetic differentiation within the PCG regions between the respective sequences.

The RelTime chronogram (Figure 4) establishes a temporal framework for the evolutionary history of the superfamily Tineoidea. Our molecular dating estimates that the MRCA of the Tineoidea lineages analyzed in this study originated approximately 87.60 Ma during the Late Cretaceous. Within the family Gracillariidae, the divergence time between the *Conopomorpha* and *Caloptilia* was dated to approximately 77.23 Ma.

Notably, the divergence time between the Taiwanese specimen (this study) and the reference *C. sinensis* sequence (OK310517) were estimated to be <0.01 Ma (indicated as 0.00 on the chronogram). This indicates an extremely high level of genetic conservation across the 13 mitochondrial PCGs, suggesting that the overall 5.35% genomic divergence (primarily driven by non-coding regions), the functional coding sequences of these geographically distinct samples have remained virtually identical since their last common ancestor. Furthermore, the divergence of the *C. sinensis* clade from its relative *Ca. theivora* occurred in the Late Cretaceous, highlighting a long-standing evolutionary trajectory independent of other gracillariid lineages.

### 3.6. Identification of Mitogenomic Divergence Region

To further clarify the 5.35% genomic divergence, a high-resolution variant analysis was performed. Only 2 single nucleotide polymorphisms (SNPs) across the entire 17,301 bp mitogenome (one was found in a tRNA and another was found in the control region) were identified. In contrast, 228 indel events were detected in whole mitogenome of Taiwan *C. sinensis*, while 90.35% (206/228) of the indel was identified within the control region. This demonstrates that the divergence is predominantly driven by structural variation in non-coding regions rather than nucleotide substitution (Supplementary Table S3).

## 4. Discussion

This study reports the complete mitogenome of *C. sinensis* from Taiwan and compares it with the previously published mitogenome (GenBank: OK310517). Both mitogenomes exhibit a rearranged tRNA cluster (*trnR-trnA-trnN-trnS1-trnE-trnF*), deviating from the typical lepidopteran order (*trnA-trnR-trnN-trnS1-trnE-trnF*). This specific rearrangement can be explained by the Duplication/Random Loss (DRL) model, where tandem duplication of tRNA genes occurs during mtDNA replication, followed by the random deletion of redundant paralogs. The presence of this rare pattern in *C. sinensis*, as well as in *Parasa consocia* (Limacodidae) and *Somatina indicataria* (Geometridae) [57,58,59], may a potential case of convergent evolution or a broader distribution of this feature across diverse Lepidopteran lineages; however, this hypothesis is needed for further validation through broader taxonomic sampling across diverse families.

The Taiwan sample shares 94.65% overall sequence identity and 99.35% identity in the PCGs with the reference. The high conservation of PCGs (99.35%) suggests that these genes are under stringent purifying selection to maintain essential mitochondrial respiratory functions [60]. Conversely, the observed 5.35% overall genomic divergence reveals substantial intraspecific variation within *C. sinensis*. Our divergence-time estimate further corroborated this relationship, yielding a split time of <0.01 Ma (represented as 0.00 Ma) between the two samples.

The negligible temporal divergence in protein-coding regions suggests that the separation between these samples is evolutionarily recent, a finding that aligns with historical records of the introduction of litchi and longan to Taiwan. The contrast between the significant structural divergence (5.35%) and the negligible temporal distance (<0.01 Ma) indicates that genetic variation is partitioned non-uniformly across the mitogenome. While the PCGs remain stable due to functional constraints, the accumulation of mutations in the control region and tRNA spacers highlights the rapid onset of genetic drift or founder effects within the island sample following geographic isolation. The negligible divergence inferred from our RelTime analysis confirms that these genetic variations, although structurally prominent in non-coding areas, have not yet translated into significant evolutionary distance within the coding sequences.

Despite this overall coding stability, notable differences were identified in the codon usage of *COX1* and *ND6*, including the variation in the *COX1* stop codon (TAA vs. T) and *ND6* start codon (ATT vs. ATA). These micro-evolutionary shifts in initiation and termination signals may influence translational efficacy or RNA stability, reflecting potential adaptive fine-tuning to local environmental conditions in geographically distinct samples. Although the current study is limited by a single regional sample, the high-quality hybrid assembly (Illumina and ONT) provided here rectifies the limitations of previous records—which lacked precise locality and raw data—and thereby significantly enhances our understanding of *C. sinensis* mitogenomics. This traceable and reliable reference offers a valuable resource for future research on molecular evolution, phylogeography, and targeted pest management strategies within the Gracillariidae family.

Additionally, the mitogenome of the Taiwan sample showed a high A + T content (83.64%), slightly higher than the reference (83.52%) and above the Lepidopteran average (80.49% ± 0.95%) [61]. Across insect orders, the range of A + T% varies, from 64% in termites to 86.7% in bees [62]. The expansion of the *C. sinensis* mitogenome to 17,301 bp is largely attributed to four prominent AT-rich intergenic spacers. Three of these are situated within the rearranged tRNA cluster, where between *trnR*/*trnA* (169 bp), *trnN*/*trnS1* (240 bp) and *trnE*/*trnF* (190 bp), while another is located between *trnS2*/*ND1*(328 bp). The length and position variation in these regions reflect significant structural divergence and suggest that these intergenic spacers may serve as hotspots for genomic instability and reorganization [63].

The observed 5.35% mitogenomic divergence between the Taiwan sample and the reference genome (OK310517) reveals substantial intraspecific variation within *C. sinensis*. Our divergence-time estimate further corroborated this relationship, yielding a split time of <0.01 Ma between the two samples. Given that litchi and longan were historically introduced to Taiwan, this divergence may stem from genetic drift or founder effects [64,65] followed by a relatively short period of geographic isolation. The negligible temporal divergence in protein-coding regions (<0.01 Ma) suggests that the separation between these samples is evolutionarily recent, consistent with the historical records of host plant introduction. The accumulation of mutations in the control region and tRNA spacers might be the preliminary evidence suggesting possible genetic differentiation. While the current analysis is limited by a single sampling from this region, it is insufficient to fully demonstrate how genetic drift manifests across the entire mitogenome at a population level or to definitively establish phylogeographic differentiation. The high-quality hybrid assembly (Illumina and ONT) provided here rectifies the limitations of previous records that lacked precise locality and raw data and thereby enhances our understanding of *C. sinensis* mitogenomics. This traceable and reliable reference offers a valuable resource for future research on the molecular evolution, phylogeography, and targeted pest management strategies regarding the Gracillariidae family.

## 5. Conclusions

The complete mitochondrial genome of *C. sinensis* from Taiwan was successfully characterized, revealing a typical gene composition but a distinct tRNA rearrangement pattern. Phylogenetic analysis robustly clustered the Taiwan specimen within the *C. sinensis* sample (OK310517), exhibiting 94.65% overall identity, while the PCGs remained highly conserved at 99.35%. Divergence time estimation indicated an evolutionary split of <0.01 Ma between the studied lineages, providing strong temporal evidence that supports a relatively recent expansion or introduction event, likely associated with host plant movement. The observed genetic differentiation, primarily localized in non-coding regions and synonymous codon usage, highlights the onset of genetic drift in the island sample despite functional stability in coding sequences.

These findings provide crucial genetic insights into the Taiwan specimen of *C. sinensis* and contribute valuable, high-quality genomic resources. This study serves as a foundational reference for further research on the evolutionary dynamics, molecular diagnostics, and biodiversity of the Gracillariidae family.

## Figures and Tables

**Table 1:** The mitogenomes are used for phylogeny reconstruction.

Name	Start	Stop	Strand	Length	Anti-Codon	Start Codon	Stop Codon	Overlapping (bp)	nt_identity (%)
*trnM*	1	68	+	68	CAT	−	−	−	100
*trnI*	69	133	+	65	GAT	−	−	5	100
*trnQ*	137	205	−	69	TTG	−	−	5	100
*ND2*	259	1272	+	1014	-	ATT	TAG	−	100
*trnW*	1273	1339	+	67	TCA	−	−	8	100
*trnC*	1332	1397	−	66	GCA	−	−	8	100
*trnY*	1398	1463	−	66	GTA	−	−	−	100
*COX1*	1466	3001	+	1536	-	CGA	TAA	5	99.6
*trnL2*	2997	3061	+	65	TAA	−	−	5	100
*COX2*	3062	3746	+	685	−	ATG	T	3	99.6
*trnK*	3744	3814	+	71	CTT	−	−	3	100
*trnD*	3815	3881	+	67	GTC	−	−	−	100
*ATP8*	3882	4040	+	159	−	ATT	TAA	−	100
*ATP6*	4034	4711	+	678	−	ATG	TAA	1	100
*COX3*	4711	5497	+	787	−	ATG	T	1	98.5
*trnG*	5510	5575	+	66	TCC	-	−	−	100
*ND3*	5576	5929	+	354	−	ATT	TAA	2	100
*trnR*	5928	5995	+	68	TCG	−	−	2	100
*trnA*	6165	6231	+	67	TGC	−	−	−	100
*trnN*	6232	6297	+	66	GTT	−	−	−	100
*trnS1*	6538	6603	+	66	GCT	−	−	−	91.4
*trnE*	6604	6671	+	68	TTC	−	−	−	94.2
*trnF*	6862	6925	−	64	GAA	−	−	−	100
*ND5*	6929	8641	−	1713	−	ATT	TAG	−	98.6
*trnH*	8666	8731	−	66	GTG	−	−	−	100
*ND4*	8735	10,070	−	1336	−	ATG	T	1	99.8
*ND4L*	10,070	10,357	−	288	−	ATG	TAA	1	100
*trnT*	10,362	10,427	+	66	TGT	−	−	−	100
*trnP*	10,428	10,492	−	65	TGG	−	-	−	100
*ND6*	10,519	11,025	+	507	−	ATT	TAA	1	95.5
*CYTB*	11,025	12,176	+	1152	−	ATG	TAA	1, 2	100
*trnS2*	12,175	12,239	+	65	TGA	−	-	2	100
*ND1*	12,568	13,503	−	936	−	ATG	TAA	−	100
*trnL1*	13,505	13,576	−	72	TAG	−	−	−	97.3
*rrnL*	13,603	14,864	−	1262	−	−	−	−	94.5
*trnV*	14,920	14,985	−	66	TAC	−	−	1	100
*rrnS*	14,985	15,754	−	770	−	−	−	1	99.1
D-Loop	15,755	17,301		1547	−	−	−	−	−

## Data Availability

The sequencing raw data were deposited in the NCBI (https://www.ncbi.nlm.nih.gov, accessed on 16 May 2025) database with the Sequence Read Archive (SRA) accession number PRJNA1263815. The mitogenome data are available in the NCBI database under the GenBank reference number PV603655.1.

## Supplementary Materials

The following supporting information can be downloaded at: https://www.mdpi.com/article/10.3390/genes17050594/s1, Figure S1: Bioinformatics workflow for genomic data preprocessing and quality control. The pipeline outlines the comparative processing streams for Illumina short reads and ONT long reads. Short-read raw data were processed for adapter removal and quality filtering using TrimGalore, with subsequent quality validation performed via FastQC. Concurrently, ONT raw signals in POD5 format were subjected to high-accuracy (HAC) basecalling, integrated demultiplexing, and adapter trimming via Dorado v7.3.11. Summary statistics for the filtered clean long reads were then generated using NanoPlot; Figure S2: Coverage plot of assembled *C*. *sinensis* mitogenome; Table S1: The cytochrome c oxidase subunit 1 gene sequences are used for phylogeny reconstruction; Table S2: The mitogenomes are used for phylogeny reconstruction; Table S3: Distribution of nucleotide substitutions (SNPs) and structural variations (indels) across genomic regions.
