# Supplementary figures and images for: The Complete Mitochondrial Genome of Conopomorpha sinensis (Lepidoptera: Gracillariidae) Sample from Taiwan
Source: Genes (Basel). 2026 May 21;17(5):594. doi: 10.3390/genes17050594 (PMC13205447; doi:10.3390/genes17050594)

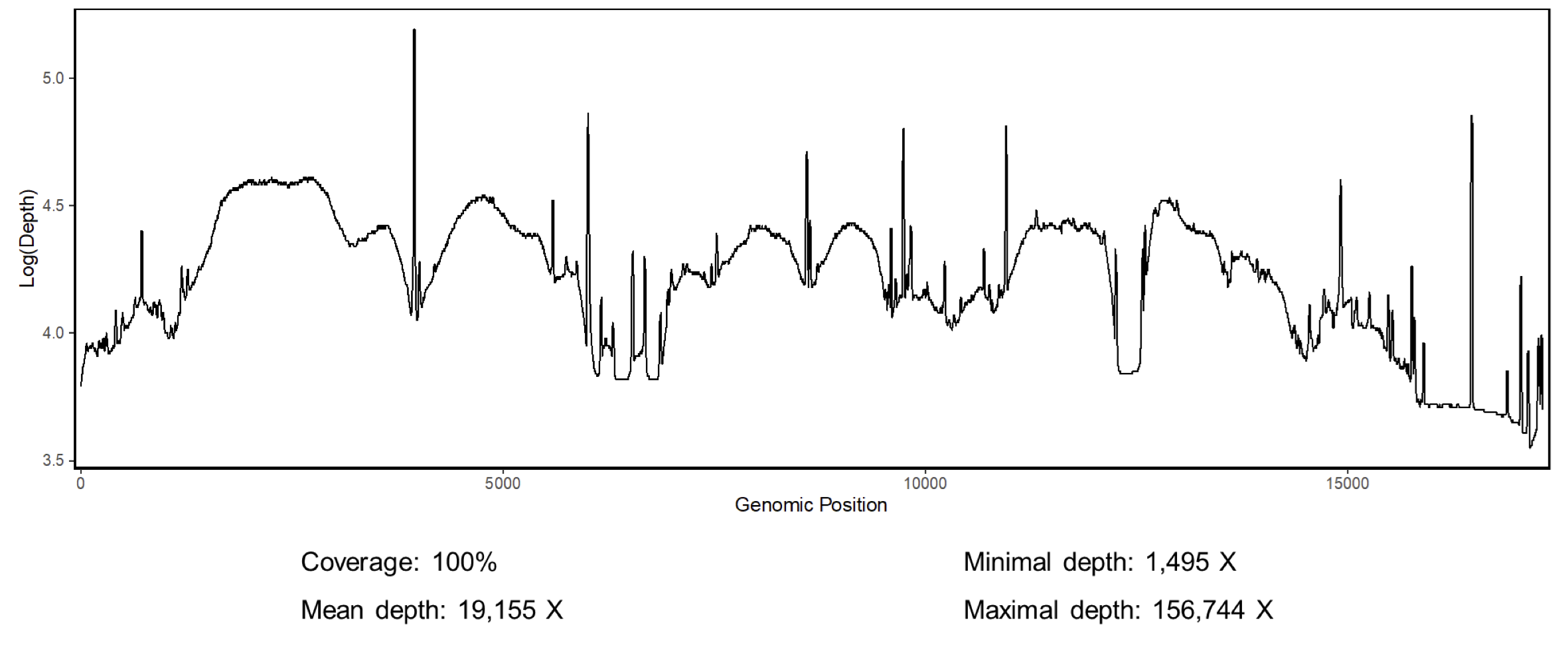

Supplement: Supplementary file 1 [file genes-17-00594-s001.zip › Supplementary Figure S2.tiff]

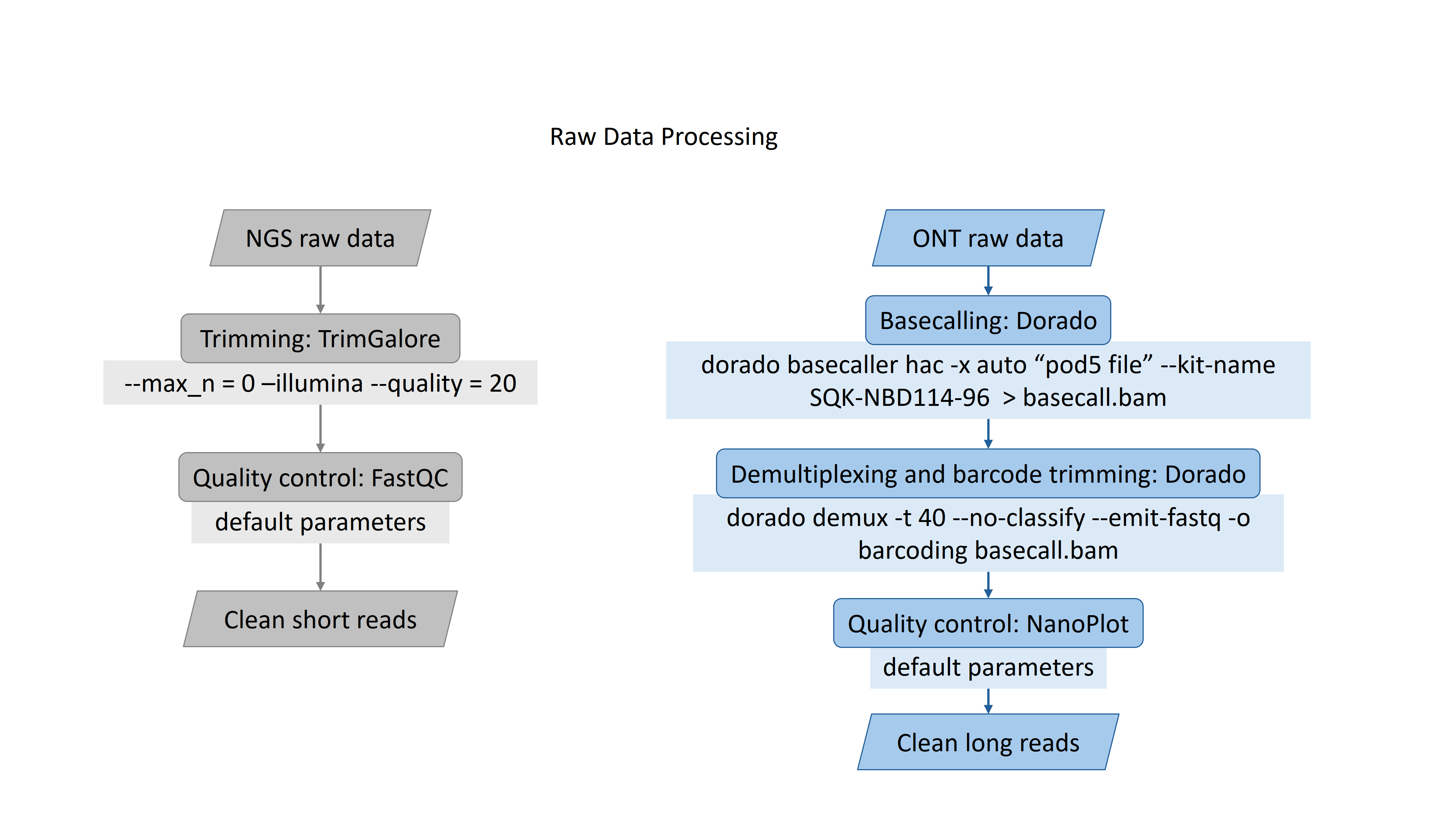

Supplement: Supplementary file 1 [file genes-17-00594-s001.zip › Supplementary Figure S1.tif]
